# Divergent foraging strategies during incubation of an unusually wide-ranging seabird, the Murphy’s petrel

**DOI:** 10.1007/s00227-018-3451-7

**Published:** 2018-12-15

**Authors:** Thomas A. Clay, Steffen Oppel, Jennifer L. Lavers, Richard A. Phillips, M. de L. Brooke

**Affiliations:** 1 British Antarctic Survey, Natural Environment Research Council, High Cross, Madingley Road, Cambridge, CB3 0ET UK; 2 Department of Zoology, University of Cambridge, Downing Street, Cambridge, CB2 3EJ UK; 3 School of Environmental Sciences, University of Liverpool, Liverpool, L69 3GP UK; 4 RSPB Centre for Conservation Science, Royal Society for the Protection of Birds, David Attenborough Building, Pembroke Street, Cambridge, CB2 3EZ UK; 5 Institute for Marine and Antarctic Studies, University of Tasmania, 20 Castray Esplanade, Battery Point, Hobart, TAS 7004 Australia

## Abstract

Divergent foraging strategies may emerge within a population due to a combination of physiological and environmental factors; yet to persist, neither strategy should offer a consistent selective advantage over the alternative in the long term. Murphy’s petrels *Pterodroma ultima* from Henderson Island (24°20′S, 128°20′W) in the South Pacific Ocean are highly vagile, and exhibit two distinct foraging trip types during incubation; similar proportions of birds undertake either looping trips around the South Pacific Gyre to waters off Peru (hereafter “East”) or trips south-west of the colony towards the Subtropical Front (“South”) (mean maximum ranges of c. 3800 or 2000 km from the colony, respectively). However, the relative benefits of the distinct trip types remain unclear. Through tracking birds with GPS and salt-water immersion loggers in 2015, the fine-scale foraging behaviour was examined for East (trip durations: 14.1–19.8 days, maximum ranges 2387–4823 km) and South trips (12.9–25.8 days, 1565–1991 km). Data on behaviour classified from GPS tracks, the number of wet bouts per hour (a proxy for landing rates) and wind speeds, were used to distinguish two distinct foraging modes: birds on East trips spent more time in directed movement, whereas those on South trips spent a greater proportion of time in area-restricted search (ARS) behaviour. East trips were associated with higher overall mass gain, and wet bouts occurred in equal proportions during directed movement and ARS behaviour. This suggests that in unproductive marine environments, it may be more profitable to maximise area covered to increase the chances of encountering prey. Analysis of lower-resolution geolocator data (collected from 2011 to 2014) indicated that individuals were largely consistent in trip type between years. Since birds that conducted East trips were 19% lighter on departure from the colony and experienced more frequent tailwinds on foraging trips, we speculate that these birds may benefit from reduced movement costs, whilst also experiencing reduced competition for foraging opportunities.

**Electronic supplementary material:**

The online version of this article (10.1007/s00227-018-3451-7) contains supplementary material, which is available to authorized users.

## Introduction

Understanding the foraging decisions of animals is a fundamental aim of ecology (Stephens and Krebs 1986). Individuals are expected to make optimal choices about where to forage to maximise energy intake, while minimising energy expenditure searching for and capturing prey (MacArthur and Pianka 1966; Pyke 1984). A variety of foraging strategies may exist within a population, reflecting a combination of physiological, behavioural and environmental drivers (McNamara and Houston 2008). Divergent strategies may result from intrinsic differences or individual specialisations (Baylis et al. 2015; Phillips et al. 2017) and may simply be alternative means of achieving, on average, the same outcome, just as groups of animals may distribute themselves between food sources according to the ideal free distribution to achieve equal foraging success (Fretwell and Lucas 1970). However, over long time periods, consistent use of particular strategies may have important implications for individual fitness and population dynamics (Bolnick et al. 2003).

Pelagic seabirds are well adapted for foraging in highly dynamic and heterogeneous marine environments (Lack 1968; Ashmole 1971; Weimerskirch 2007) and often cover vast areas to find food (Spear and Ainley 1998; Ballance and Pitman 1999; Weimerskirch et al. 2003, 2005). During the breeding season, birds are energetically constrained by having to return to the colony to incubate the egg or feed the chick, and foraging strategies may vary according to a suite of factors, including the degree of constraint imposed by breeding and the availability of foraging habitat (Phillips et al. 2009, 2017). The natural rate of breeding failure may be higher during incubation than during chick-rearing (Prince et al. 1994), likely because parents have to restore body condition as well as accumulate energy for the next fast on the nest (Chastel et al. 1995; Weimerskirch 1995). A trade-off exists between the two members of the pair whereby prolonged foraging by one partner will deplete the energy stores of the other to the extent that they may desert the egg and the breeding attempt fails (Chaurand and Weimerskirch 1994; Cleeland et al. 2014). Short foraging trips, therefore, reduce the risk of nest desertion (Johnstone and Davis 1990). In contrast, longer trips may provide birds with greater foraging opportunities and enable them to restore body reserves (Weimerskirch 1995). For seabirds which have to travel further to find food, the duration of trips is likely to be longer, such that trip duration and maximum foraging range tend to be closely linked (Weimerskirch 2007). Indeed, both are generally linked to the predictability and availability of resources, indicating an environmental constraint on foraging strategies; species which feed in more predictable and less distant habitats such as shelf breaks or upwellings have shorter foraging trips than oceanic and tropical species (Lewis et al. 2001; Weimerskirch 2007).

Gadfly petrels *Pterodroma* spp. have particularly long incubation shifts, averaging 13–19 days, thought to be related to their reliance on oceanic habitats (Warham 1990; Brooke 2004). In many species, males and females conduct just one and two foraging trips, respectively (Johnstone and Davis 1990; Brooke 1995), but relatively little is known about their foraging ecology. Some of the longest incubation trips known are undertaken by Murphy’s Petrels *Pterodroma ultima* from Henderson Island (24°20′S, 128°20′W) in the central South Pacific Ocean (Brooke 1995; Oppel et al. 2018). Indeed, a recent study found that adults spend c. 95% of their time in flight and travel remarkable distances to find food, with trip durations of up to 29 days and a maximum range of almost 5000 km, likely due to the low productivity of marine habitats around the breeding colony (Clay et al. 2017). Two distinct foraging trip types were identified: looping trips ranging c. 3800 km to the north-east, or more directed trips ranging c. 2000 km to the south or south-west of Henderson Island. Murphy’s petrels are monomorphic and unlike many species with sexual size dimorphism (Shaffer et al. 2001a; Phillips et al. 2004), differences in foraging strategies among individuals appear to be unrelated to sex (Clay et al. 2017). Murphy’s petrels have a low rate of mass loss (relative to body size) while incubating the egg (Brooke 1995), which may extend their threshold for egg desertion beyond that of other species (Johnstone and Davis 1990; Chaurand and Weimerskirch 1994). Nonetheless, we would expect that longer or more distant trips would result in measurable benefits such as greater mass gain, as observed in other procellariiform species (e.g. Weimerskirch 1995; Kim et al. 2017). However, so far, the ecological drivers and consequences of these two distinct strategies remain poorly understood.

In this study, we tracked Murphy’s petrels concurrently with GPS and immersion loggers to investigate their fine-scale foraging behaviour and the trade-offs associated with the different trip types. Specifically, through the use of a behavioural clustering algorithm applied to GPS tracks and the number of wet bouts derived from immersion loggers, we aimed to determine whether (1) at-sea activity patterns differed between the two trip types. We also linked movement and wind data to (2) determine if more distant foraging trips were assisted by tail winds, which presumably reduce travel costs. Additionally, we assessed (3) whether foraging trip type was linked to body mass, and (4) whether birds on more distant trips gained more mass at sea, which would indicate overall higher foraging success. Finally, to determine whether foraging decisions might be related to longer-term specialisations, we revisited a multi-year geolocator dataset collected by Clay et al. (2017) to examine (5) whether individual birds consistently used the same foraging strategy in different years.

## Materials and methods

### Device deployment and retrieval

Fieldwork took place in July 2011, 2013 and 2015 on Henderson Island where c. 2500 pairs of Murphy’s petrels breed (Brooke 1995). We tracked adult birds breeding amongst the vegetation along North Beach. In 2011, 25 geolocator-immersion loggers (1.9 g; Mk18H, British Antarctic Survey, Cambridge, UK) were deployed on incubating or brooding birds; 18 were retrieved in 2013 (see Clay et al. 2017 for further details) and one more in 2015. In 2015, FastLoc GPS devices (10 g; PathTrack, Yorkshire, UK) and immersion loggers (Intigeo-C65: 14 × 8 × 6 mm and 1 g; Migrate Technology Ltd, Cambridge, UK) were deployed on 10 incubating birds, of which three had previously been tracked with geolocators. GPS devices were attached to the four central tail feathers using Tesa^®^ tape and programmed to obtain a GPS position every 40 min. Immersion loggers were attached to a plastic ring on the tarsus. The total mass of the GPS device, immersion logger, rings and attachment materials (c. 12 g) was around the 3% body mass limit recommended for pelagic seabirds (Phillips et al. 2003), based on an average mass of 400 g (Brooke 1995). All birds were handled for < 20 min and returned to their nest upon release. Once devices had been attached, the nest was checked every 1–3 days to determine departure and return dates, and to retrieve the attached loggers. During device deployment, all but two birds were weighed with a Pesola spring balance and wing length (from the wrist joint to the tip of the longest primary feather) was measured by SO and JL, but to minimise disturbance, they were not handled again prior to departure. All birds were then weighed again when first encountered after their return to the nest. We were unable to assess the influence of handling and tagging on breeding success, because chicks at all monitored nests (*n* = 57, including unmarked control birds) were killed by rats *Rattus exulans* within 5 days of hatching (Lavers et al. 2016). Birds were not sexed since previous work found no sex differences in foraging distributions during incubation (Clay et al. 2017).

### Data processing

GPS data were first filtered to remove locations at the nest and foraging trips were defined to last > 2.5 h and extend > 3 km from the colony (Oppel et al. 2015). Three trips were incomplete due to logger battery capacity; for these trips, arrival at the colony was derived from nest observations (i.e. the date the tracked birds were first seen back on their nests). As nests were not monitored daily, the return dates were calculated using the mean difference between arrival dates derived from GPS data and nest observations for birds whose complete trip was recorded. We alternatively inferred return dates from the immersion data (based on the timing of the last wet bout), but because differences between the two methods were negligible we only present return dates based on GPS and nest monitoring data. We also calculated the following metrics: the cumulative distance travelled between all locations assuming straight-line Euclidean distances (km), the maximum distance from the colony (km; hereafter “maximum range”), and the average travel speed (km h^−1^). In addition, for all trips, we calculated the departure direction of foraging trips as the great circle route bearing between the colony and the first location 24 h after departure in the package circular (Lund et al. 2017). Results did not differ when we selected a range of cut-offs (6, 12, 18, and 36 h after departure). For each trip, mass gain was calculated for each bird by subtracting the departure mass from the return mass. Both departure and return mass were estimated from mass at weighing and the time difference between capture and departure or return and capture, assuming a constant rate of mass loss of 5.25 g day^−1^ (Brooke 1995). Initially, after prey ingestion, birds are likely to have a higher rate of mass loss due to absorption of water content, but the aqueous phase is emptied from the stomach within 12 h (Roby et al. 1989), and so is unlikely to influence our results.

At-sea behaviour during foraging trips was quantified using data from multiple sensors: (1) speed and tortuosity from GPS tracks, indicating whether a bird displayed area-restricted search (ARS) behaviour (Kareiva and Odell 1987), and (2) wet bouts from immersion logger data that indicated whether a bird was in contact with the water surface. The predominant feeding method of Murphy’s petrels is surface seizing, which involves eating dead or alive prey after landing on the sea surface (Spear et al. 2007); thus, a wet bout is likely to indicate a feeding attempt, given that breeding birds appear to spend a negligible (< 5%) amount of time resting at sea (Clay et al. 2017). Behaviour classification was based on two input variables: instantaneous travel speed and turning angles between subsequent locations, using Expectation Maximisation binary Clustering (EMbC; Garriga et al. 2016), an algorithm based on Gaussian Mixture Model maximum likelihood estimation which requires few prior assumptions and has captured biologically meaningful behaviours in a range of species (Louzao et al. 2014; Garriga et al. 2016). It uses the distributions of the two input variables to partition locations and identify thresholds for slow and fast movements, and for low (shallow angles) and high (wide angles) values of turning. Wide turning angles between locations are generally considered to be associated with ARS behaviour, while high speeds and straight tracks are typical of fast, directed movement (Garriga et al. 2016; de Grissac et al. 2017; Diop et al. 2018). We differentiated between four different behaviours, namely directed movement (high speed, shallow angles), resting on the water (low speeds, shallow angles; inclusive of sit-and-wait feeding), and intensive (low speeds, wide angles) and extensive searching (high speeds, wide angles) (see Table S1 in supplementary material) (Louzao et al. 2014; Garriga et al. 2016). We consider intensive and extensive search modes to represent small- and large-scale ARS, respectively (Weimerskirch et al. 2007).

Immersion loggers tested for saltwater immersion every 30 s, storing the sum of positive tests (between 0 and 10) at the end of each 5-min period. A flight bout was defined as at least one continuous 5-min period spent entirely dry (0) and a wet bout as a 5-min period when at least one wet event was recorded. We considered the following activity metrics as indicators of foraging and flight behaviour: the proportion of time spent on the water (wet), the average duration of flight (dry) bouts in minutes and the number of wet bouts (succeeding dry events) as a proxy for the number of landings. We acknowledge that the low sampling interval is likely to underestimate landing rates (Johnson et al. 2017). However, omission errors are likely to be similar across individuals and trip types.

The average duration of flight bouts was extracted for each trip and the proportion of time spent on the water calculated as the sum of positive tests divided by the total number of tests. To plot activity in space, we linked GPS and activity data by summing the number of wet bouts in the 20 min before and after each location. As activity patterns vary diurnally (Clay et al. 2017), each location was categorised as daylight or darkness using civil twilight as a cut-off (i.e. when the sun is 6° below the horizon). The number of wet bouts was averaged by individual and day, and summarised by trip type to visualise temporal patterns, particularly at the start and end of trips (see Fig. S1). Data from one immersion logger were unavailable due to battery failure.

### Statistical analysis

We categorised foraging trips as “East” or “South” based on non-overlapping differences in maximum ranges (Clay et al. 2017, Table 1, Fig. S2). Visualisation of maximum ranges for each trip over time revealed that at around 5 days into the trip, the distances from the colony of birds on South trips had reached an asymptote, whereas distances continued to increase for East trips (Fig. S2a). Using these simple criteria, we were also able to categorise incomplete trips into one of the two types. For cumulative distance travelled and average travel speed, only values for complete trips are reported. For maximum range, we present the values from all except one trip, where the GPS battery failed while the individual was at its furthest point from the colony (Table 1). We constructed generalised linear models (GLMs) to test for differences with trip type in the trip duration, number of wet bouts per hour, proportion of time spent on the water, average flight bout duration, wing length, departure and return mass and mass gain (both in g and as a percentage of the departure mass). Maximum range, cumulative distance travelled and average travel speed were not compared statistically, as the first was used to differentiate the two trip types, and sample sizes for the latter two variables were too low because data were unavailable for incomplete trips. Response variables had a Gaussian distribution except for trip duration which had a Poisson distribution. The proportion of time on the water and proportional mass gain were logit transformed. The significance of the covariate trip type was assessed using likelihood ratio tests (LRTs, Zuur et al. 2009). Departure directions of East and South trips were compared using a circular analysis of variance in the circular package (Lund et al. 2017). We also ran separate GLMs to test the effect of maximum range on departure mass, return mass, and overall mass gain.

**Table 1:** Comparison of trip characteristics, mass gain and activity patterns of Murphy’s petrels *Pterodroma ultima* from Henderson Island tracked with GPS and immersion loggers in 2015, by trip type

	South	East	Statistic	*p*
Trip metrics				
Trip duration (days)	18.6 ± 6.1 (5)	17.4 ± 2.6 (5)	$\chi_{1}^{2}$ = 0.3	0.603
Maximum range (km)	1840 ± 190 (4)	3729 ± 889 (5)	–	–
Cumulative distance (km)	8137 ± 498 (2)	13,053 ± 2380 (5)	–	–
Average travel speed (km h^−1^)	35.4 ± 11.3 (2)	31.1 ± 2.7 (5)	–	–
Departure direction (°) over first 24 h	242 ± 24 (5)	223 ± 26 (5)	*F*_1_= 1.4	0.264
Body mass				
Departure mass (g)	405 ± 59 (4)	341 ± 28 (4)	$\chi_{1}^{2}$ = 3.9	**0.047**
Return mass (g)	435 ± 44 (5)	412 ± 17 (5)	$\chi_{1}^{2}$ = 1.4	0.238
Mass gain (g)	48 ± 22 (4)	74 ± 15 (4)	$\chi_{1}^{2}$ = 3.8	**0.049**
Mass gain (% of dep. mass)	12 ± 7 (4)	22 ± 6 (4)	$\chi_{1}^{2}$ = 3.9	**0.048**
Activity patterns				
No. wet bouts (h^−1^)	0.85 ± 0.04 (5)	0.95 ± 0.06 (4)	$\chi_{1}^{2}$ = 8.6	**0.003**
Time spent on water (%)	9 ± 4 (5)	8 ± 2 (4)	$\chi_{1}^{2}$ = < 0.1	0.993
Average flight bout duration (min)	63.5 ± 4.8 (5)	56.1 ± 5.5 (4)	$\chi_{1}^{2}$ = 1.5	0.216

Data are means ± standard deviations, with sample sizes in parentheses. Test statistics are provided with a *p* value, and significant differences between the trip types shown in bold. Sample sizes varied for the following reasons: trip metrics = maximum range, cumulative distance travelled and average travel speed were unreliable for one, three and three incomplete trips, respectively; body mass = two birds not measured on deployment of devices; activity patterns = one logger failed to record activity; however, activity for trips for which GPS data were incomplete was still used as immersion loggers lasted the duration of trips

We compared the amount of time spent in different behaviours, as derived from the EMbC algorithm, between trip types, and between daylight and darkness periods. A multivariate analysis of variance (MANOVA) was run with the proportion of time spent in the four behaviours as the response variable, and with trip type and day vs. night as covariates. Proportions were logit transformed, with the significance of covariates tested using Pillai’s trace tests. Univariate ANOVAs were subsequently carried out to test for covariate effects on individual behaviours. We compared outputs from multiple sensors, i.e. we tested whether wet bouts derived from immersion loggers were associated with ARS behaviour, classified by the EMbC algorithm. To determine whether wet bouts occurred more often in particular behavioural modes, we calculated the proportion of wet bouts in each trip that occurred during each behaviour (Table 2), and compared the number of wet bouts per hour in each behaviour using a linear model including the covariates trip type, behaviour, and their interaction.

**Table 2:** The percentage of (a) time spent in different behaviours by Murphy’s petrels *Pterodroma ultima* from Henderson Island conducting South and East trips, split by daylight (day) and darkness (night) periods, and (b) wet bouts in different behaviours for daylight and darkness periods combined

Behaviour	South		East	
	Day	Night	Day	Night
(a) Percentage of time				
Extensive search	24 ± 7	20 ± 9	14 ± 5	8 ± 3
Intensive search	14 ± 4	15 ± 6	4 ± 3	6 ± 3
Resting	11 ± 3	12 ± 4	8 ± 3	9 ± 3
Directed movement	51 ± 10	53 ± 12	74 ± 10	77 ± 8
(b) Percentage of wet bouts				
Extensive search	23 ± 6		12 ± 6	
Intensive search	16 ± 6		7 ± 5	
Resting	12 ± 4		8 ± 1	
Directed movement	49 ± 8		74 ± 10	

Behaviours were determined from GPS tracks using the Expectation Maximisation binary Clustering algorithm, and wet bouts from immersion loggers. Values are the means of individual values ± standard deviation. See Table S2 in supplementary material for statistical details

Additionally, wind speed and direction from zonal (*U*) and meridional (*V*) wind components were obtained from the National Centres for Environmental Prediction (NCEP)/National Centre for Atmospheric Research (NCAR) Reanalysis dataset at a 6-h temporal resolution and were associated with each GPS position via the R-package RNCEP (Kemp et al. 2012). Tailwind was calculated as the component of the flow parallel to the direction of movement for each location. We used two-sided Kolmogorov–Smirnov tests to test for significant differences between the cumulative frequency distribution of tailwinds encountered by birds on South and East foraging trips, only considering locations associated with intensive and extensive search and directed movement.

### Repeatability of foraging strategies between years

Using incubation trips from birds tracked for 2 + years with geolocators (see Clay et al. 2017 for details on general population-level patterns and data processing), we investigated whether individuals adopted the same strategy (i.e. South or East trips) in different years. We initially fitted a hierarchical state-space model to trips from all individuals to better correct observed locations for geolocation errors (Jonsen et al. 2003; Carneiro et al. 2016). As there are no published estimates of geolocator error in gadfly petrels, we used a fixed error of 1.66° and 1.82° for latitude and longitude, respectively, based on previous double-tagging studies in pelagic seabirds (Phillips et al. 2004; Winship et al. 2012). The state-space model was run using Markov Chain Monte Carlo (MCMC) sampling in the bsam package (Jonsen et al. 2013). Two chains of 5000 samples from the joint posterior probability distribution were obtained after a burn-in of 100,000, retaining every 20th of the remaining samples to reduce autocorrelation. Convergence was assessed visually by checking trace, density and autocorrelation plots.

**Fig. 1:**
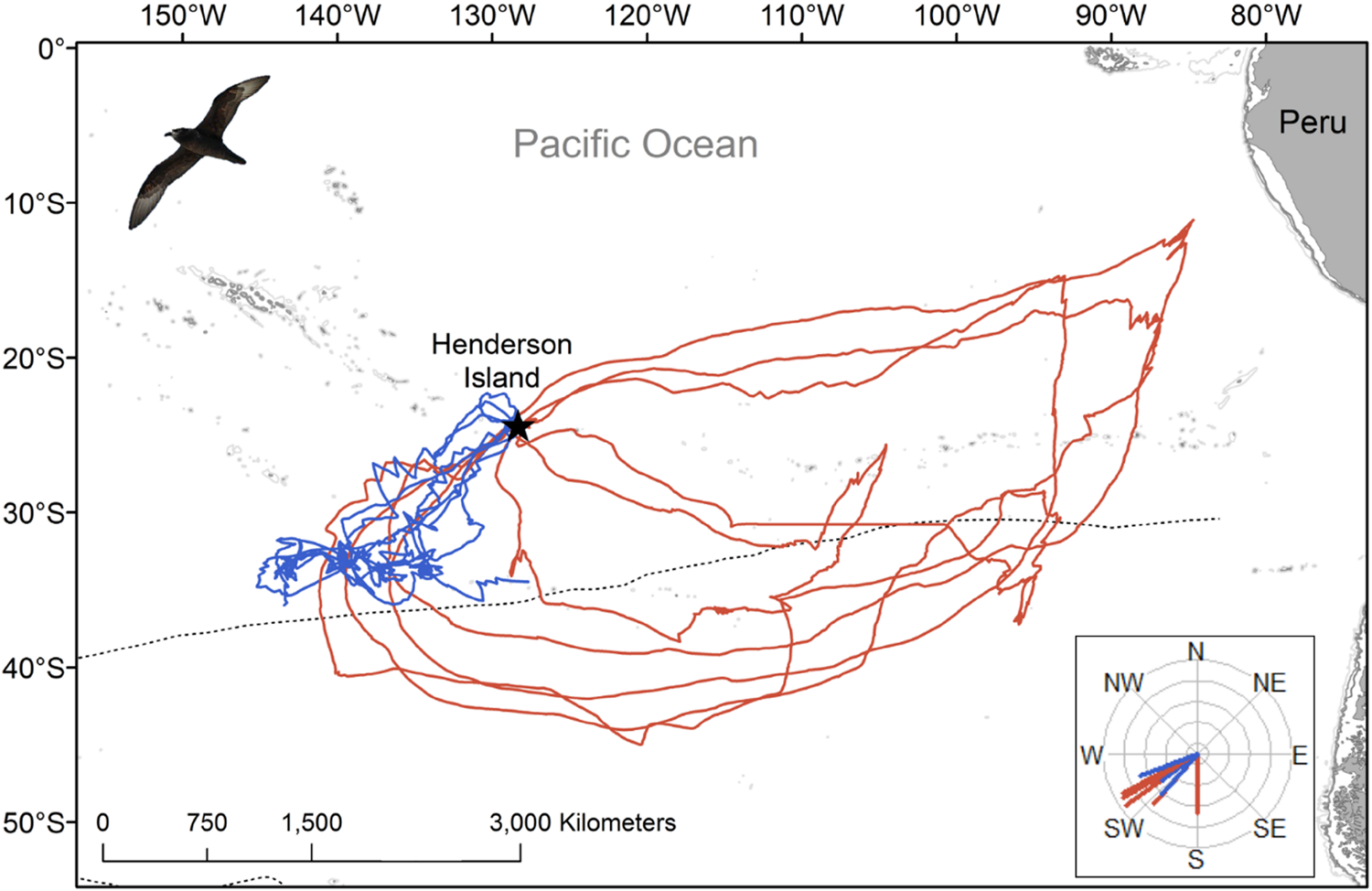
GPS tracks of East (red, *n* = 5) and South (blue, *n* = 5) incubation foraging trips undertaken by Murphy’s petrels *Pterodroma ultima* breeding on Henderson Island (black star), Pitcairn Islands, in the South Pacific Ocean. The departure bearing of each trip over the first 24 h is shown, as inset; all East trips followed the same counter-clockwise travel pattern. The position of the Subtropical Front is indicated by a dotted line and the 500, 1000 and 2000 m isobaths are shown with grey lines. Three South trips are incomplete due to logger battery failure. All tracks are interpolations between subsequent GPS fixes; for one East trip there was a period of signal loss represented by an unusually straight line

As East and South trips differed greatly in the location of the furthest point (distal location; Fig. 1, Clay et al. 2017), we also calculated the bearing of this point from the colony as a simple proxy for trip type. We calculated the repeatability (*r*, a measure ranging from 0 = low to 1 = high) of the trip duration, cumulative travel distance and maximum distance from the colony, their associated standard errors and *p* values in the rptR package (Nakagawa and Schielzeth 2010), to test the null hypothesis that between-individual variance in each metric was equal to the within-individual variance. For the bearing to the distal location, we used a circular ANOVA (in the circular package) and calculated repeatability manually using mean squared error (Lessells and Boag 1987). A *p* value was not available for this test (see Patrick et al. 2014 for details). As few individuals were tracked for > 1 trip in any given year, analyses were conducted on just the first trip. Using all trips (from both GPS devices and geolocators) we ran Spearman rank correlations to determine if foraging strategies differed within the incubation period, i.e. if there was a link between departure date and trip characteristics (trip duration, maximum range and cumulative distance travelled). All analyses were conducted in R v. 3.3.1 (R Core Team 2014) and unless otherwise reported, data are presented as a mean ± standard deviation.

## Results

Ten birds were tracked with GPS loggers during incubation in 2015 and all but three (all of which were South trips) yielded complete foraging trips. The three incomplete trips yielded GPS data for only around half of their duration, but immersion data were available for the whole duration of these trips. The tracked birds adopted one of the two distinct foraging strategies (East or South trips); five birds used a region south-west of the colony, just north of the Subtropical Front, while five birds took more distant, looping trips eastwards, following an anti-clockwise pattern (Fig. 1). All but one bird departed the colony during daylight between 05:39 and 18:16 local time (mean 15:05), but return times showed no obvious diurnal pattern, i.e. were in daylight or darkness (01:35–23:56). One individual departed on a foraging trip, returned to the colony for around 22 h, then departed again; we considered this to be one trip, as this colony visit was not long enough to relieve the partner. While we could not be sure whether trips were the first or second incubation trips conducted by individuals, analysis of trip characteristics (from all complete GPS and geolocator trips) found no effect of calendar date (trip duration: *r*_s_ = − 0.24, *S* = − 18,847, *p *= 0.110; maximum range *r*_s_ = 0.07, *S* = 14,120, *p *= 0.649; cumulative distance travelled: *r*_s_ = 0.09, *S* = 13,774, *p *= 0.545), suggesting little population-level variation in foraging strategies across the incubation period.

### Comparison of time allocation between South and East trips

South trips were similar in duration to East trips (South = 18.6 ± 6.1 days, range 12.9–25.8 days; East = 17.4 ± 2.6 days, range 14.1–19.8 days; $\chi_{1}^{2}$ = 5.4, *p* = 0.3; Table 1); however, birds conducting East trips ranged further from the colony (South = 1840 ± 190 km, range 1565–1991 km; East = 3729 ± 889 km, range 2387–4823 km; Table 1), with a greater cumulative distance travelled (for complete trips; South = 8137 ± 498 km, range 7784 –8489 km; East = 13,053 ± 2380 km, range 9104–14,695 km; Table 1), yet had a fairly similar average travel speed (for complete trips; South = 35.4 ± 11.3 km h^−1^, East = 31.1 ± 2.7 km h^−1^; Table 1). Both South and East trips took an initial south-westerly bearing (Fig. 1, Table 1); however, birds conducting East trips continued southwards, crossing the Subtropical front before heading eastwards towards South America.

**Fig. 2:**
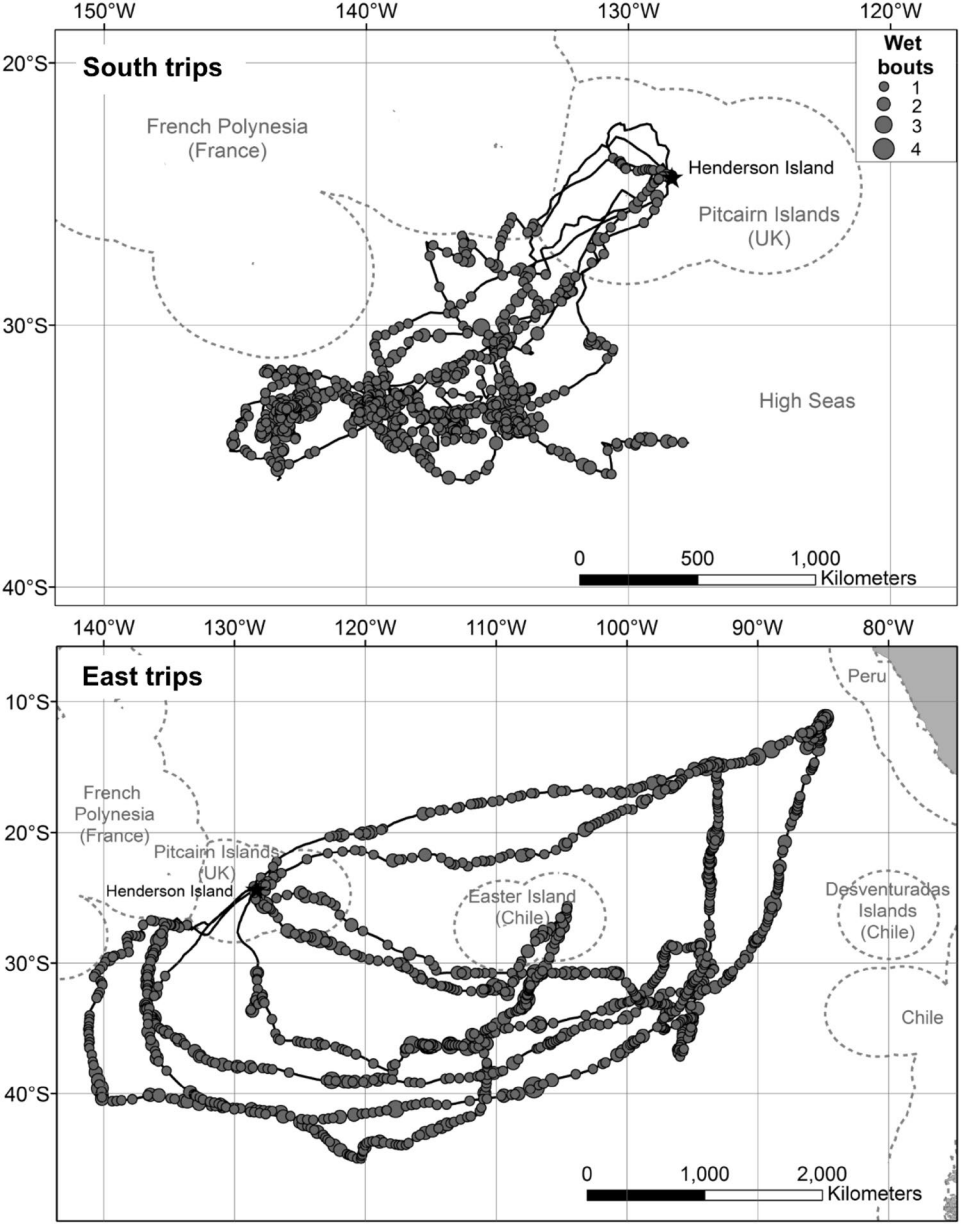
The spatial distribution of wet bouts associated with South (*n* = 5) and East (*n* = 4) foraging trips of Murphy’s petrels *Pterodroma ultima*, from combined tracking with GPS and immersion loggers in 2015. Wet bouts were summed for the period 20 min either side of each location, and are shown as grey circles which vary in size according to the number. Exclusive Economic Zones of countries in the South Pacific Ocean are shown with dotted grey lines. Three South tracks are incomplete due to logger battery failure

Birds accomplished a greater number of wet bouts per hour on East than South trips (Table 1); however, the proportion of time spent on the water and duration of flight bouts did not differ between trip types (Table 1). There was no evidence of rafting behaviour at the start of the trip; indeed, we did not record any wet bouts during the first c. 20 h of either trip type, indicating that petrels were also unlikely to be actively capturing prey during this period, unless these capture events were extremely short and consistently fell between the 30 s sampling rate of the immersion logger. There was a fairly even number of wet bouts per hour from the second day until the last few days of the trip, at which point there appeared to be a slight reduction in the number of wet bouts until the end of the trip (Fig. S1). Apart from the first day, Murphy’s petrels appeared to land on the water throughout trips with no concentration of effort in a particular area or during daylight or darkness (Fig. 2). The petrels spent only 9% and 15% of their time within the Pitcairn Islands Exclusive Economic Zone (EEZ), a recently declared marine reserve (Avagliano et al. 2015; Risotto 2015) during East and South trips, respectively. An even smaller proportion of wet bouts (4% for both trip types) was made in the reserve area, with the majority occurring in international waters (Fig. 2).

**Fig. 3:**
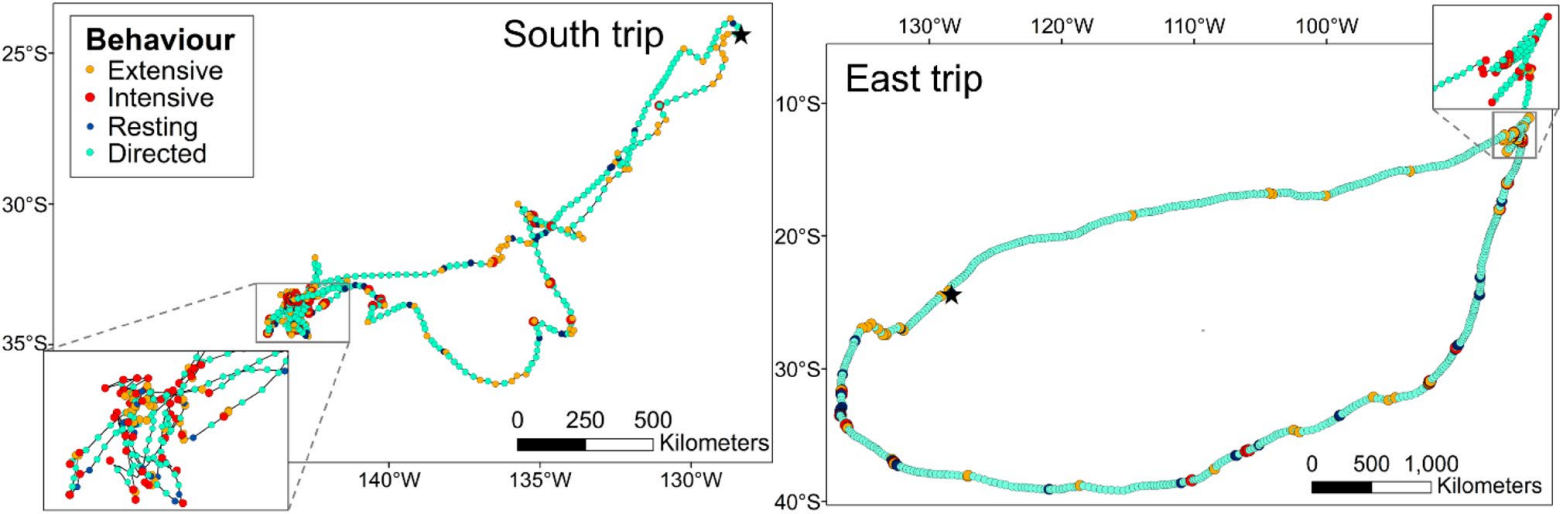
Example trips of each type (South and East) with each location coloured by behavioural modes assigned by EMbC: extensive and intensive search, resting and directed movement. Insets are segments of trips where a greater proportion of intensive search behaviour took place. The location of Henderson Island is shown by a black star. Note that the scales of the two plots differ

**Fig. 4:**
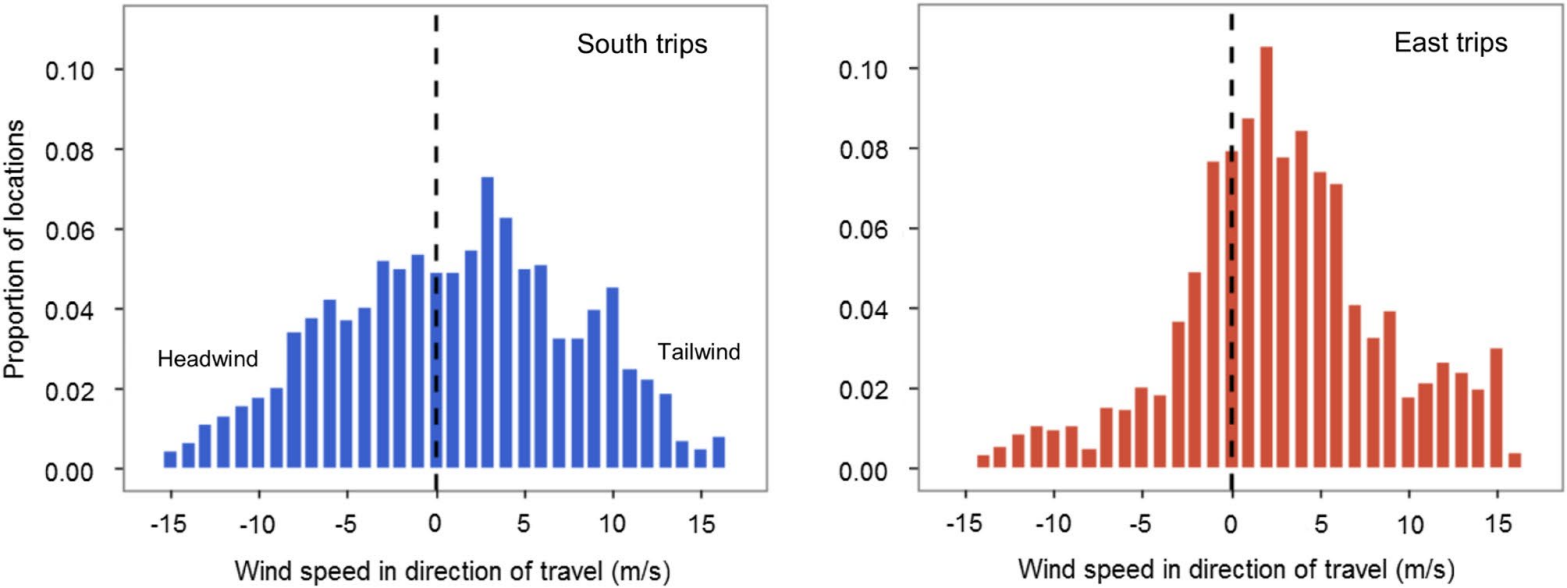
Relative frequency histograms of wind speeds relative to the direction of travel of Murphy’s petrels tracked with GPS loggers in 2015, on South (blue) and East (red) trips

The proportion of time spent in different behaviours (classified from movement characteristics) differed with trip type (*F*_1_ = 8.0, *p* = 0.001), but not day vs. night (*F*_1_ = 2.4, *p* = 0.102). A much greater proportion of time spent on East (> 70%) than South trips (c. 50%) was classified as directed movement, and correspondingly smaller proportions of time were spent in ARS behaviours (intensive and extensive search; East = c. 15–20%, South = c. 35–40%; Tables 2, S2, Fig. 3). For both trip types, intensive and extensive search occurred both by day and night, but a slightly greater proportion of time was spent in extensive search during daylight (Tables 2, S2). There was no difference in the average hourly number of wet bouts achieved by birds engaged in the four behaviours (behaviour: trip type = $\chi_{1}^{2}$ = 1.1, *p* = 0.776; behaviour = $\chi_{1}^{2}$ = 4.5, *p* = 0.606). As a result, the allocation of wet bouts to different behaviours closely reflected their overall time budgets (Table 2). Birds experienced proportionally more tailwinds on East than South trips (*D*-statistic = 0.2, *p* < 0.001; Fig. 4), likely by following a route along predictable trade winds, particularly on return journeys.

### Mass gain

**Fig. 5:**
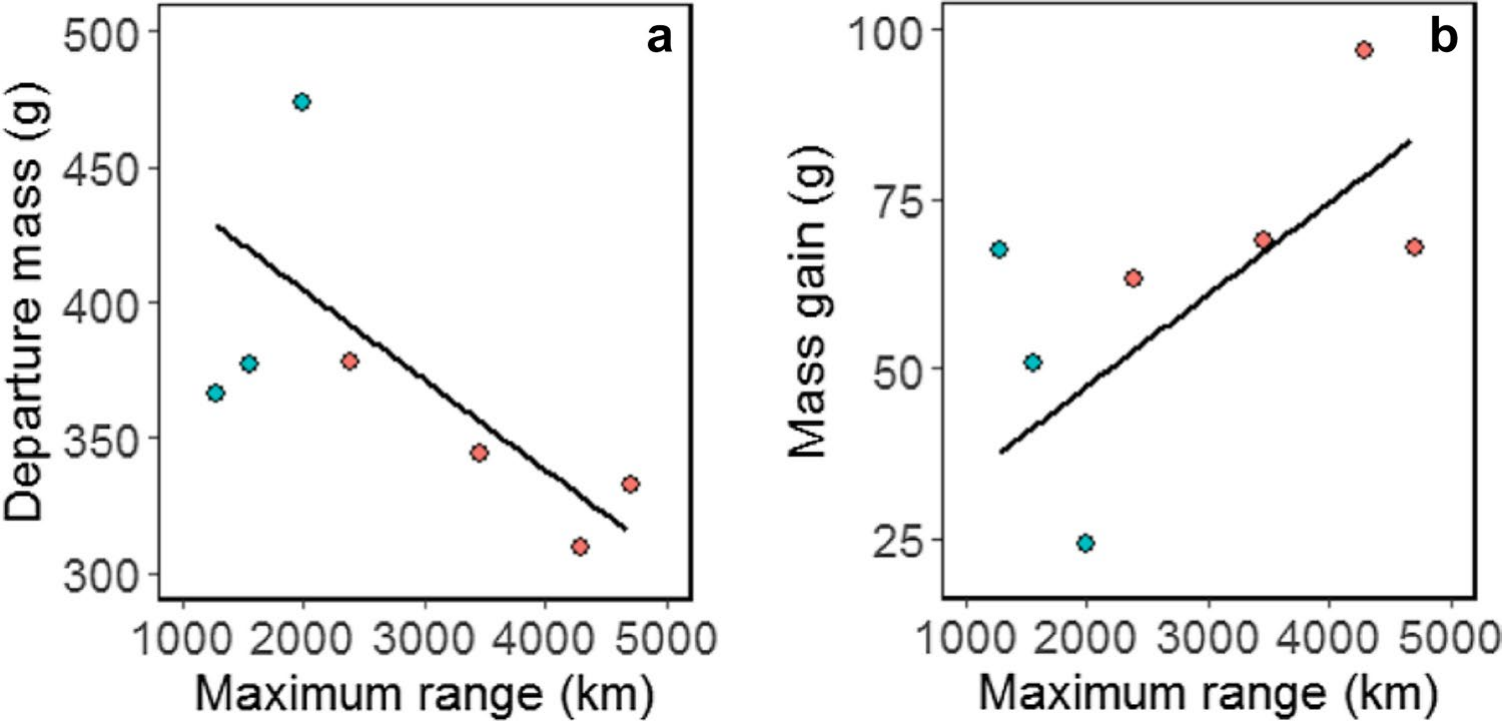
The relationship between maximum range (km) and **a** departure mass and **b** total mass gain (g) of Murphy’s petrels *Pterodroma ultima* tracked with GPS loggers in 2015. South and East trips are indicated by blue and red dots, respectively, and the modelled relationship is shown with a black line. The sample size is reduced as two birds were not weighed before departure, and one incomplete trip was removed as the bird was at its furthest point from the colony when the logger battery failed

Birds conducting East trips had 18.8% lower mass at departure (East = 341 ± 28 g; South = 405 ± 59 g; $\chi_{1}^{2}$ = 3.9, *p* = 0.047; Table 1) but a fairly similar mass upon return to the colony (East = 412 ± 17 g; South = 435 ± 44 g; $\chi_{1}^{2}$ = 1.4, *p* = 0.238). Wing length did not differ (East = 28.7 ± 0.5 cm; South = 28.5 ± 0.2 cm; $\chi_{1}^{2}$ = 1.2, *p* = 0.270), indicating little structural difference between birds conducting East and South trips. Birds that were lighter at departure were more likely to travel further from the colony ($\chi_{1}^{2}$ = 4.7, *p* = 0.029; Fig. 5a), and those birds that travelled further also gained more mass ($\chi_{1}^{2}$ = 4.5, *p* = 0.034; Fig. 5b), because there was no relationship between return mass and travel distance ($\chi_{1}^{2}$ = 1.9, *p* = 0.167). Consequently, birds on East trips had slightly greater mass gain in absolute terms (East = 74 ± 15 g; South = 48 ± 22 g; $\chi_{1}^{2}$ = 3.8, *p* = 0.049; Table 1), but almost double the percentage of their departure mass compared to birds on South trips (East = 22 ± 6%; South = 12 ± 7%; $\chi_{1}^{2}$ = 3.9, *p* = 0.048; Table 1).

### Repeatability of trip types

**Fig. 6:**
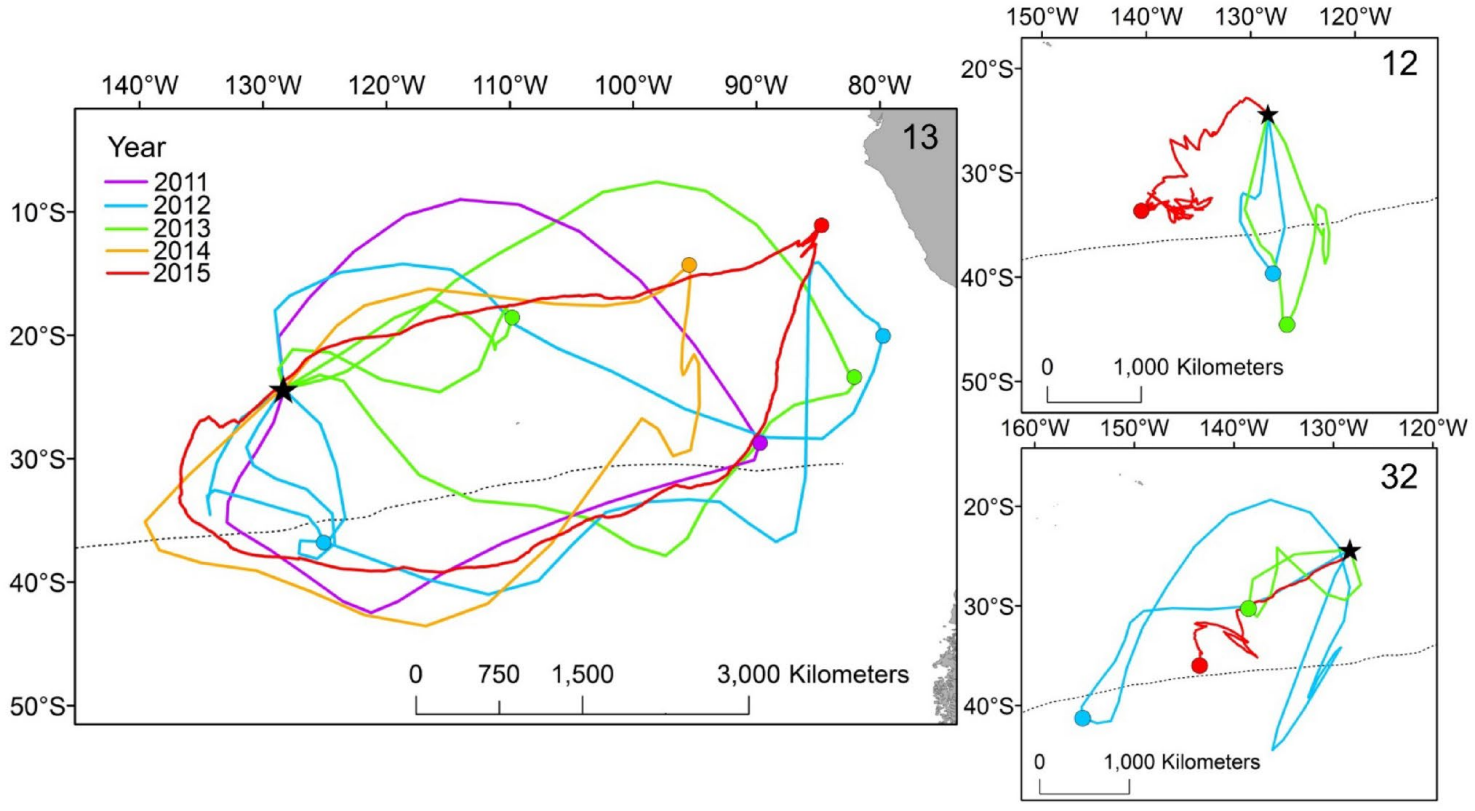
Repeat foraging trips of three individual Murphy’s petrels *Pterodroma ultima* (individual identities 12, 13 and 32) tracked with geolocators (2011–2014) and GPS loggers (2015): ID 13 = 7 trips over 5 years (2011–2015), ID 12 = 3 trips over 3 years (2012, 2013 and 2015) and ID 32 = 3 trips over 3 years (2012, 2013 and 2015). Distal locations of trips are shown by coloured dots and the position of the Subtropical Front is shown by a dotted line. Two red GPS trips are incomplete due to logger battery failure

Repeat incubation trips (*n* = 32) were recorded for 13 individuals tracked with geolocators between 2011 and 2014, mostly in two consecutive seasons. Inclusive of GPS data, two individuals were tracked for 3 trips (in 3 years) and one individual for 7 trips (in 5 years). These three appeared to be largely consistent in their foraging trip type (South or East) among years (Fig. 6). There was relatively high repeatability of foraging trip types as determined by the bearing from the colony to the distal locations of foraging trips (*r* = 0.58 ± 0.17) (Fig. S3). We also found moderate repeatability in trip durations (*r* = 0.49 ± 0.21, *p* = 0.029) but not in the maximum ranges (*r* = 0.35 ± 0.21, *p* = 0.126) nor in the cumulative distances travelled (*r* = 0.21 ± 0.20, *p* = 0.300).

## Discussion

We analysed data on movement and activity patterns, and changes in body mass, to quantify the potential benefits associated with the two contrasting foraging strategies of incubating Murphy’s petrels. GPS tracking revealed that while South and East trips were of a similar duration, the furthest point reached on anticlockwise looping East trips was much further from the colony than on the more directed South trips. By combining the GPS data with geolocator data from multiple years, we show that the relative proportion of birds conducting South and East trips was consistent across years, and within individuals, and therefore appears to be largely independent of environmental conditions experienced by foraging birds, at least in our short time-series. We found that East trips were generally undertaken by lighter birds and associated with higher overall mass gain, suggesting that trip type may be linked to intrinsic attributes such as body mass, while the benefits of conducting more distant looping trips appear to outweigh those related to foraging closer to the colony.

Unlike many species breeding in tropical environments which disperse in multiple directions (Hennicke and Weimerskirch 2014; Oppel et al. 2015; Mott et al. 2016), all the tracked Murphy’s petrels initially departed in a similar direction. Birds did not appear to land on the water during the first 20 h, suggesting they neither raft nor feed intensively in waters within a few hundred km of the colony, which are some of the least productive on Earth (Claustre and Maritorena 2003). However, because the sampling interval of immersion loggers was low (every 30 s), it is possible that short prey-capture events could have been missed. Since we recorded a substantial number of wet bouts within the last 24 h of trips as birds were approaching the colony, the absence of wet activity at the beginning of trips is likely unrelated to the recording resolution. Nevertheless, we found that a negligible proportion of wet activity (< 5% of bouts) occurred within the Pitcairn Islands no-take reserve, which covers the Pitcairn Islands Exclusive Economic Zone (EEZ) (Avagliano et al. 2015; Risotto 2015); this emphasises the challenges associated with the at-sea protection of wide-ranging marine predators such as gadfly petrels (Lavers et al. 2014; Clay et al. 2017; Ramos et al. 2017; Oppel et al. 2018).

The foraging behaviour of seabirds is considered to reflect the availability and predictability of their prey (Weimerskirch 2007). That all South trips targeted a region south-west of the colony suggests that at a large spatial scale (100s km), this region has elevated prey density. Indeed, when the petrels reached waters associated with the Subtropical Front, more than 750 km away (Clay et al. 2017), tracks became more sinuous, which can indicate ARS behaviours (Kareiva and Odell 1987) associated with regions of higher and more predictable prey density (Fauchald and Tveraa 2003; Weimerskirch et al. 2007). Behavioural time allocation differed between trip types; birds that remained south of the colony spent c. 35–40% of their time in ARS behaviour, whereas those on East trips allocated more time (c. 75%) to directed movement. However, our results may have been influenced to some degree by GPS logger battery failure for several South trips. In contrast to South trips, the looping East trips of Murphy’s petrels did not appear to target a particular region, but the birds likely reduced energetic costs associated with travel by using tailwinds, namely westerlies at 35°–40°S and predictable anticyclonic trade winds at the edges of the South Pacific Gyre. These trips are analogous to those conducted by wandering albatrosses *Diomedea exulans* when feeding on oceanic resources (Weimerskirch et al. 1997), reinforcing previous suggestions that for seabirds which feed in regions where resources are likely to be patchy or unpredictable, one of the most effective strategies is to travel large distances in a continuous search for prey, while using prevailing winds to minimize movement costs (Ballance and Pitman 1999; Weimerskirch et al. 2000, 2005).

For both trip types, wet bouts did not appear to be clustered, suggesting regular feeding across a trip on prey that exhibit little spatial aggregation. Although short feeding events may have been missed by the 30-s resolution of the immersion logger, we consider it unlikely that foraging hotspots would have been missed solely because of the recording resolution. Indeed, we linked saltwater-immersion activity with behavioural modes derived from EMbC and found that the number of wet bouts conducted per hour was similar, regardless of the behavioural mode, with the majority occurring during bouts of directed movement. As the resolutions of both the GPS and immersion data are relatively coarse, it is unclear which of the two behavioural classification methods provides the better measure of foraging activity. Nonetheless, our results suggest that while Murphy’s petrels do conduct ARS, the majority of prey search and capture appears to take place in long directed movement phases. That birds make regular landings without conducting stereotypic ARS behaviour emphasises that it may not always be suitable to infer foraging modes from behavioural classification of step lengths and turning angles alone (Bennison et al. 2017).

During incubation, there is a trade-off for seabirds between the potential benefit of increasing the duration or distance travelled during a foraging trip to improve their own body condition, and the associated cost of depleting the body reserves of their partner, which could ultimately lead to nest desertion (Chaurand and Weimerskirch 1994). While we found no differences in trip duration between the two trip types, the maximum range of East trips was twice that of South trips. Despite our small sample size, we found that maximum range was positively related to mass gain, which indicates that the more distant, looping trips may be more profitable than South trips. However, we cannot discount the possibility that birds conducting different types of trip consume prey with different energy density, which might render differences in body mass misleading. However, given that birds on looping trips to the East had potentially more time during the return journey to the colony to digest and convert watery prey into stomach oils, we consider it unlikely that different states of digestion between individuals would have distorted our results.

Our findings that lighter birds travel further from the colony, and the more distant trips are associated with a greater number of landings support previous studies of procellariiformes such as wandering albatrosses and northern giant petrels *Macronectes halli*, (Weimerskirch 1995; González-Solís et al. 2000; Shaffer et al. 2001b). It could be suggested that South trips might entail less risk to birds as their reduced range from the colony enables them to return more quickly if foraging success is high. However, in the absence of data on foraging success (mass of prey caught per hour), the greater number of wet bouts and high mass gain associated with more distant trips suggests that their prey encounter may be higher (Tveraa et al. 1997; Weimerskirch et al. 2005), but that this may come at the cost of further travel. Nevertheless, birds on East trips are likely to profit for several reasons. Firstly, the more dispersed distributions of birds on looping trips east likely reduce competition with conspecifics, in comparison to South trips (Ballance et al. 1997). Secondly, South trips were associated with more sinuous ARS behaviour, which can be energetically costly (Amélineau et al. 2014). Moreover, it has been suggested that a smaller body size might be adaptive for travelling large distances at little cost (Barbraud et al. 1999; Yamamoto et al. 2015), particularly in areas of the tropics with relatively light winds (Spear and Ainley 1998). As there was little difference in wing length between birds conducting East and South trips, the lower body mass of birds on East trips is likely to result in increased flight performance.

As all birds initially travelled in a similar direction, it is possible that the decision of which type of trip to take is made after a few days of travel, depending on a bird’s own condition, flight costs, and foraging success en route (Phillips et al. 2009). For example, birds might pass through the Subtropical Convergence Zone, and make the decision whether to stay and search for prey or continue on a more dispersive, looping trip. Additionally, differences in mass between birds conducting East and South trips suggest that foraging strategies may be linked to intrinsic differences that may persist over longer time-scales, such as differences in intrinsic quality (Lescroël et al. 2010), or individual specialisation irrespective of these factors (Phillips et al. 2017). Indeed, we found that the propensity of individuals to conduct South or East trips is more consistent between years than expected by chance, even though some individuals do switch between two trip types both within and between years. While our analyses were restricted by the small number of trips per bird, it is particularly challenging to obtain multiple trips for a given individual when incubation stints are so long. Nonetheless, the consistency in trip metrics is somewhat surprising for a species which breeds in the tropics and forages in an unproductive oceanic environment (Weimerskirch 2007; Ceia and Ramos 2015). However, repeatability in foraging strategy does not necessarily indicate fidelity to foraging areas, as among-individual variance was very high; the distal points of trips of the same individual were often several thousands of kilometres apart.

The initial decision about when to return is likely to be influenced by the mass of the incubating partner (Tveraa et al. 1997), and so the profitability of a foraging strategy may ultimately depend on an individual’s mate. Unfortunately, we have no information on the mass, trip duration or foraging strategy of the mates of our tracked birds. Future work should investigate the factors influencing the long-term persistence of these strategies, as well as the movement patterns of birds from other colonies, and the extent to which members of the pair regulate foraging trips and body mass throughout the incubation period, according to their own condition and that of the mate (Weimerskirch 1995; Tveraa et al. 1997).

## Conclusion

In this study, we document two clear foraging trip types with associated differences in foraging behaviour, likely related to differences in marine habitats targeted. Our results suggest that looping trips to the east may be more profitable, as birds are able to maximise the area covered in search of prey, while benefiting more from favourable winds. As individuals consistently performed similar trip types in different years, we speculate that trip type may be influenced by intrinsic attributes such as body mass, as light birds might be better adapted for long-distance travel in calmer, tropical areas. We acknowledge our conclusions are limited by the small number of individuals tracked with GPS loggers; nonetheless, this study provides novel insights into the fine-scale foraging behaviour of gadfly petrels, including how they balance their foraging requirements in particularly unproductive environments. Also, due to their extremely large foraging range, which results in them spending < 20% of their time on foraging trips within the Pitcairn Islands Marine Reserve, we suggest that Murphy’s petrels are unlikely to be amenable to protection using area-based measures, and will require broader conservation measures at appropriately large scales (Oppel et al. 2018).

## Electronic supplementary material

Below is the link to the electronic supplementary material.

Supplementary material 1 (DOCX 721 kb)

## References

[CR2] Amélineau F, Péron C, Lescroël A, Authier M, Provost P, Grémillet D (2014). Windscape and tortuosity shape the flight costs of northern gannets. J Exp Biol.

[CR3] Ashmole NP, Farner DS, King JR (1971). Seabird ecology and the marine environment. Avian biology.

[CR4] Avagliano E, Bocquet A, Kape J (2015). Pitcairn Islands Ecosystem Profile.

[CR5] Ballance LT, Pitman RL (1999) Foraging ecology of tropical seabirds. In: Proceedings of the 22nd international ornithological congress, Durban. BirdLife South Africa

[CR6] Ballance LT, Pitman RL, Reilly SB (1997). Seabird community structure along a productivity gradient: importance of competition and energetic constraint. Ecology.

[CR7] Barbraud C, Weimerskirch H, Robertson GG, Jouventin P (1999). Size-related life history traits: insights from a study of snow petrels (*Pagodroma nivea*). J Anim Ecol.

[CR8] Baylis AMM, Orben RA, Arnould JPY, Peters K, Knox T, Costa DP, Staniland IJ (2015). Diving deeper into individual foraging specializations of a large marine predator, the southern sea lion. Oecologia.

[CR9] Bennison A, Bearhop S, Bodey TW, Votier SC, Grecian WJ, Wakefield ED, Hamer KC, Jessopp M (2017). Search and foraging behaviors from movement data: a comparison of methods. Ecol Evol.

[CR10] Bolnick DI, Svanback R, Fordyce JA, Yang LH, Davis JM, Hulsey CD, Forister ML (2003). The ecology of individuals: incidence and implications of individual specialization. Am Nat.

[CR11] Brooke MdL (1995). The breeding biology of the gadfly petrels *Pterodroma* spp. of the Pitcairn Islands: characteristics, population sizes and controls. Biol J Linn Soc.

[CR12] Brooke MdL (2004). Albatrosses and petrels across the world: procellariiformes.

[CR13] Carneiro APB, Manica A, Clay TA, Silk JRD, King M, Phillips RA (2016). Consistency in migration strategies and habitat preferences of brown skuas over two winters, a decade apart. Mar Ecol Prog Ser.

[CR14] Ceia FR, Ramos JA (2015). Individual specialization in the foraging and feeding strategies of seabirds: a review. Mar Biol.

[CR15] Chastel O, Weimerskirch H, Jouventin P (1995). Influence of body condition on reproductive decision and reproductive success in the Blue Petrel. Auk.

[CR16] Chaurand T, Weimerskirch H (1994). Incubation routine, body mass regulation and egg neglect in the Blue Petrel *Halobaena caerulea*. Ibis.

[CR17] Claustre H, Maritorena S (2003). The many shades of Ocean Blue. Science.

[CR18] Clay TA, Phillips RA, Manica A, Jackson H, Brooke MdL (2017). Escaping the oligotrophic gyre? The year-round movements, foraging behaviour and habitat preferences of Murphy’s petrels. Mar Ecol Prog Ser.

[CR19] Cleeland JB, Lea M-A, Hindell MA (2014). Use of the Southern Ocean by breeding short-tailed shearwaters (*Puffinus tenuirostris*). J Exp Mar Biol Ecol.

[CR20] Core Team R (2014). R: a language and environment for statistical computing.

[CR21] Diop N, Zango L, Beard A, Ba CT, Ndiaye PI, Henry L, Clingham E, Oppel S, Gonzalez-Solis J (2018). Foraging ecology of tropicbirds breeding in two contrasting marine environments. Mar Ecol Prog Ser.

[CR22] Fauchald P, Tveraa T (2003). Using first-passage time in the analysis of area-restricted search and habitat selection. Ecology.

[CR23] Fretwell SD, Lucas HLJ (1970). On territorial behavior and other factors influencing habitat distribution in birds. Acta Biotheor.

[CR24] Garriga J, Palmer JRB, Oltra A, Bartumeus F (2016). Expectation-maximization binary clustering for behavioural annotation. PLoS One.

[CR25] González-Solís J, Croxall JP, Wood AG (2000). Sexual dimorphism and sexual segregation in foraging strategies of northern giant petrels, *Macronectes halli*, during incubation. Oikos.

[CR26] de Grissac S, Bartumeus F, Cox SL, Weimerskirch H (2017). Early-life foraging: behavioral responses of newly fledged albatrosses to environmental conditions. Ecol Evol.

[CR27] Hennicke JC, Weimerskirch H (2014). Coping with variable and oligotrophic tropical waters: foraging behaviour and flexibility of the Abbott’s booby *Papasula abbotti*. Mar Ecol Prog Ser.

[CR28] Johnson LR, Boersch-Supan PH, Phillips RA, Ryan SJ (2017). Changing measurements or changing movements? Sampling scale and movement model identifiability across generations of biologging technology. Ecol Evol.

[CR29] Johnstone RM, Davis LS (1990). Incubation routines and foraging-trip regulation in the Grey-faced Petrel *Pterodroma macroptera gouldi*. Ibis.

[CR30] Jonsen ID, Myers RA, Flemming JM (2003). Meta-analysis of animal movement using state-space models. Ecology.

[CR31] Jonsen ID, Basson M, Bestley S, Bravington MV, Patterson TA, Pedersen MW, Thomson R, Thygesen UH, Wotherspoon SJ (2013). State-space models for bio-loggers: a methodological road map. Deep Sea Res Part II Top Stud Oceanogr.

[CR32] Kareiva P, Odell G (1987). Swarms of predators exhibit “Preytaxis” if individual predators use area-restricted search. Am Nat.

[CR33] Kemp MU, van Loon E, Shamoun-Baranes J, Bouten W (2012). RNCEP: global weather and climate data at your fingertips. Methods Ecol Evol.

[CR34] Kim Y, Priddel D, Carlile N (2017). Incubation routine and associated changes in body mass of Gould’s Petrel (*Pterodroma leucoptera*). Emu Austral Ornithol.

[CR35] Lack D (1968). Ecological adaptations for breeding in birds.

[CR36] Lavers JL, Miller MGR, Carter MJ, Swann G, Clarke RH (2014). Predicting the spatial distribution of a seabird community to identify priority conservation areas in the Timor Sea. Conserv Biol.

[CR37] Lavers JL, McLelland GTW, Mackinnon L, Bond A, Oppel S, Donaldson AH, Duffield ND, Forrest AK, Havery S, O’Keefe S, Torr N, Warren P (2016). Henderson Island expedition report: May–November 2015.

[CR38] Lescroël A, Ballard G, Toniolo V, Barton KJ, Wilson PR, Lyver PO, Ainley DG (2010). Working less to gain more: when breeding quality relates to foraging efficiency. Ecology.

[CR39] Lessells CM, Boag PT (1987). Unrepeatable repeatabilities: a common mistake. Auk.

[CR40] Lewis S, Sherratt TH, Hamer KC, Wanless S (2001). Evidence of intra-specific competition for food in a pelagic seabird. Nature.

[CR41] Louzao M, Wiegand T, Bartumeus F, Weimerskirch H (2014). Coupling instantaneous energy-budget models and behavioural mode analysis to estimate optimal foraging strategy: an example with wandering albatrosses. Mov Ecol.

[CR42] Lund U, Agostinelli C, Arai H, Gagliardi A, Portugues EG, Giunchi D, Irisson J-O, Pocernich M, Rotolo F (2017) Package “circular”. Available at https://cran.r-project.org/web/packages/circular/index.html. Accessed 29 June 2017

[CR43] MacArthur RH, Pianka ER (1966). On optimal use of a patchy environment. Am Nat.

[CR44] McNamara JM, Houston AI (2008). Optimal annual routines: behaviour in the context of physiology and ecology. Phil Trans R Soc B.

[CR45] Mott R, Herrod A, Clarke RH (2016). Resource partitioning between species and sexes in Great Frigatebirds and Lesser Frigatebirds. Auk.

[CR46] Nakagawa S, Schielzeth H (2010). Repeatability for Gaussian and non-Gaussian data: a practical guide for biologists. Biol Rev.

[CR47] Oppel S, Beard A, Fox D, Mackley E, Leat E, Henry L, Clingham E, Fowler N, Sim J, Sommerfeld J, Weber N, Weber S, Bolton M (2015). Foraging distribution of a tropical seabird supports Ashmole’s hypothesis of population regulation. Behav Ecol Sociobiol.

[CR48] Oppel S, Bolton M, Carneiro A, Dias M, Green JA, Masello J, Owen O, Phillips R, Quillfeldt P, Beard A, Bertrand S, Blackburn J, Boersma PD, Borges A, Broderick A, Catry P, Cleasby I, Clingham E, Creuwels J, Crofts S, Cuthbert R, Dallmeijer H, Davies R, Davies D, Dilley B, Dinis H, Dossa J, Dunn M, Efe M, Fayet A, Figueiredo L, Frederico A, Gjerdrum C, Godley B, Granadeiro J, Guilford T, Hamer K, Hazin C, Hedd A, Henry L, Hernández- Montero M, Hinke J, Kokubun N, Leat E, Metzger B, Militao T, Montrond G, Mullié W, Padget O, Pearmain E, Pollet I, Puetz K, Quintana F, Ratcliffe N, Ronconi R, Ryan P, Saldanha S, Shoji A, Simac J, Small C, Soanes L, Takahashi A, Trathan PN, Trivelpiece W, Veen J, Wakefield E, Weber N, Weber S, Zango L, Daunt F, Ito M, Harris MP, Newell MA, Wanless S, González-Solís J, Croxall J (2018). Spatial scales of marine conservation management for breeding seabirds. Mar Policy.

[CR49] Patrick SC, Bearhop S, Grémillet D, Lescroël A, Grecian WJ, Bodey TW, Hamer KC, Wakefield E, Le Nuz M, Votier SC (2014). Individual differences in searching behaviour and spatial foraging consistency in a central place marine predator. Oikos.

[CR50] Phillips RA, Xavier JC, Croxall JP (2003). Effects of satellite transmitters on albatrosses and petrels. Auk.

[CR51] Phillips RA, Silk JR, Phalan B, Catry P, Croxall JP (2004). Seasonal sexual segregation in two *Thalassarche albatross* species: competitive exclusion, reproductive role specialization or foraging niche divergence?. Proc R Soc B.

[CR52] Phillips RA, Wakefield ED, Croxall JP, Fukuda A, Higuchi H (2009). Albatross foraging behaviour: no evidence for dual foraging, and limited support for anticipatory regulation of provisioning at South Georgia. Mar Ecol Prog Ser.

[CR53] Phillips RA, Lewis S, González-Solís J, Daunt F (2017). Causes and consequences of individual variability and specialization in foraging and migration strategies of seabirds. Mar Ecol Prog Ser.

[CR54] Prince PA, Rothery P, Croxall JP, Wood AG (1994). Population dynamics of Black-browed and Grey-headed Albatrosses *Diomedea melanophris* and *D. chrysostoma* at Bird Island, South Georgia. Ibis.

[CR55] Pyke GH (1984). Optimal foraging theory: a critical review. Annu Rev Ecol Syst.

[CR56] Ramos R, Carlile N, Madeiros J, Ramírez I, Paiva VH, Dinis HA, Zino F, Biscoito M, Leal GR, Bugoni L, Jodice PGR, Ryan PG, González-Solís J (2017). It is the time for oceanic seabirds: tracking year-round distribution of gadfly petrels across the Atlantic Ocean. Divers Distrib.

[CR57] Risotto A (2015). Pew, National Geographic Applaud Creation of Pitcairn Islands Marine Reserve.

[CR58] Roby DD, Brink KL, Place AR (1989). Relative passage rates of lipid and aqueous digesta in the formation of stomach oils. Auk.

[CR59] Shaffer SA, Weimerskirch H, Costa DP (2001). Functional significance of sexual dimorphism in Wandering Albatrosses, *Diomedea exulans*. Funct Ecol.

[CR60] Shaffer SA, Costa DP, Weimerskirch H (2001). Behavioural factors affecting foraging effort of breeding wandering albatrosses. J Anim Ecol.

[CR61] Spear LB, Ainley DG (1998). Morphological differences relative to ecological segregation in petrels (Family: Procellariidae) of the Southern Ocean and Tropical Pacific. Auk.

[CR62] Spear LB, Ainley DG, Walker WA (2007). Foraging dynamics of seabirds in the Eastern Tropical Pacific Ocean. Stud Avian Biol.

[CR63] Stephens DW, Krebs JR (1986). Foraging theory.

[CR64] Tveraa T, Lorensten S-H, Sæther B-E (1997). Regulation of foraging trips and costs of incubation shifts in the Antarctic petrel (*Thalassoica antarctica*). Behav Ecol.

[CR65] Warham J (1990). The petrels: their ecology and breeding systems.

[CR66] Weimerskirch H (1995). Regulation of foraging trips and incubation routine in male and female wandering albatrosses. Oecologia.

[CR67] Weimerskirch H (2007). Are seabirds foraging for unpredictable resources?. Deep Sea Res Part II Top Stud Oceanogr.

[CR68] Weimerskirch H, Wilson RP, Lys P (1997). Activity pattern of foraging in the wandering albatross: a marine predator with two modes of prey searching. Mar Ecol Prog Ser.

[CR69] Weimerskirch H, Guionnet T, Martin J, Shaffer SA, Costa DP (2000). Fast and fuel efficient? Optimal use of wind by flying albatrosses. Proc R Soc B.

[CR70] Weimerskirch H, Chastel O, Barbraud C, Tostain O (2003). Frigatebirds ride high on thermals. Nature.

[CR71] Weimerskirch H, Gault A, Cherel Y (2005). Prey distribution and patchiness: factors in foraging success and efficiency of wandering albatrosses. Ecology.

[CR72] Weimerskirch H, Pinaud D, Pawlowski F, Bost CA (2007). Does prey capture induce area-restricted search? A fine-scale study using GPS in a marine predator, the wandering albatross. Am Nat.

[CR1] Winship AJ, Jorgensen SJ, Shaffer SA, Jonsen ID, Robinson PW, Costa DP, Block BA (2012). State-space framework for estimating measurement error from double-tagging telemetry experiments. Methods Ecol Evol.

[CR73] Yamamoto T, Kohno H, Mizutani A, Yoda K, Matsumoto S, Kawabe R, Watanabe S, Oka N, Sato K, Yamamoto M, Sugawa H, Karino K, Shiomi K, Yonehara Y, Takahashi A (2015). Geographical variation in body size of a pelagic seabird, the streaked shearwater *Calonectris leucomelas*. J Biogeogr.

[CR74] Zuur A, Ieno EN, Walker N, Saveliev AA, Smith GM (2009). Mixed effects models and extensions in ecology with R.

