# Supplementary material for: Divergent foraging strategies during incubation of an unusually wide-ranging seabird, the Murphy’s petrel
Source: Mar Biol. 2018 Dec 15;166(1):8. doi: 10.1007/s00227-018-3451-7 (PMC6295288; doi:10.1007/s00227-018-3451-7)
Supplement: Supplementary file 1 — Supplementary material 1 (DOCX 721 kb) [file 227_2018_3451_MOESM1_ESM.docx]

***Electronic supplementary material***

**Divergent foraging strategies during incubation of an unusually wide-ranging seabird, the Murphy’s petrel**

Thomas A. Clay^1, 2, 3*^, Steffen Oppel^4^, Jennifer L. Lavers^5, 6^, Richard A. Phillips^1^, and M. de L. Brooke^2^

^1^ British Antarctic Survey, Natural Environment Research Council, High Cross, Madingley Road, Cambridge, UK, CB3 0ET, United Kingdom.

^2^ Department of Zoology, University of Cambridge, Downing Street, Cambridge, CB2 3EJ, United Kingdom.

^3^ School of Environmental Sciences, University of Liverpool, Liverpool, L69 3GP, United Kingdom

^4^ RSPB Centre for Conservation Science, Royal Society for the Protection of Birds, David Attenborough Building, Pembroke Street, Cambridge, CB2 3EZ, United Kingdom.

^5^ RSPB Centre for Conservation Science, Royal Society for the Protection of Birds, The Lodge, Sandy, Bedfordshire SG19 2DL, United Kingdom.

^6^ Institute for Marine and Antarctic Studies, University of Tasmania, 20 Castray Esplanade, Battery Point, Tasmania, 7004, Australia.

*Corresponding author: [tommy.clay@outlook.com](mailto:tommy.clay@outlook.com)

**Table S1**. Values of speed and turning angle thresholds for the four behaviours assigned by the Expectation Maximisation binary Clustering (EMbC) algorithm. Means ± standard deviation of thresholds are shown.

|  | Extensive search | Intensive search | Resting | Directed movement |
| --- | --- | --- | --- | --- |
| Speed (km h^-1^) | 29.99 ± 11.99 | 6.48 ± 4.28 | 6.16 ± 14.4 | 34.42 ± 10.94 |
| Turning angle (radians) | 1.56 ± 0.89 | 1.64 ± 0.80 | 0.26 ± 0.22 | 0.11 ± 0.09 |

**Table S2**. Results of the effect of trip type (South or East) and daylight or darkness and their two-way interaction on the proportion of time spent in each of the four behaviours. All behaviours were modelled together in MANOVAs but the results of univariate ANOVAs are shown to distinguish behaviour-specific differences. For each comparison, the test statistic and *p*-value are provided, and significant relationships are shown in bold.

| Behaviour | Trip type | Day *vs.* night |
| --- | --- | --- |
| Extensive search | F_1_ = 18.2, ***p* < 0.001** | F_1_ = 4.9 , ***p* = 0.040** |
| Intensive search | F_1_ = 31.2, ***p* < 0.001** | F_1_ = 1.8, *p* = 0.198 |
| Resting | F_1_ = 4.2, *p* = 0.055 | F_1_ = 0.5, *p* = 0.477 |
| Directed movement | F_1_ = 27.9, ***p* < 0.001** | F_1_ = 0.3, *p* = 0.582 |


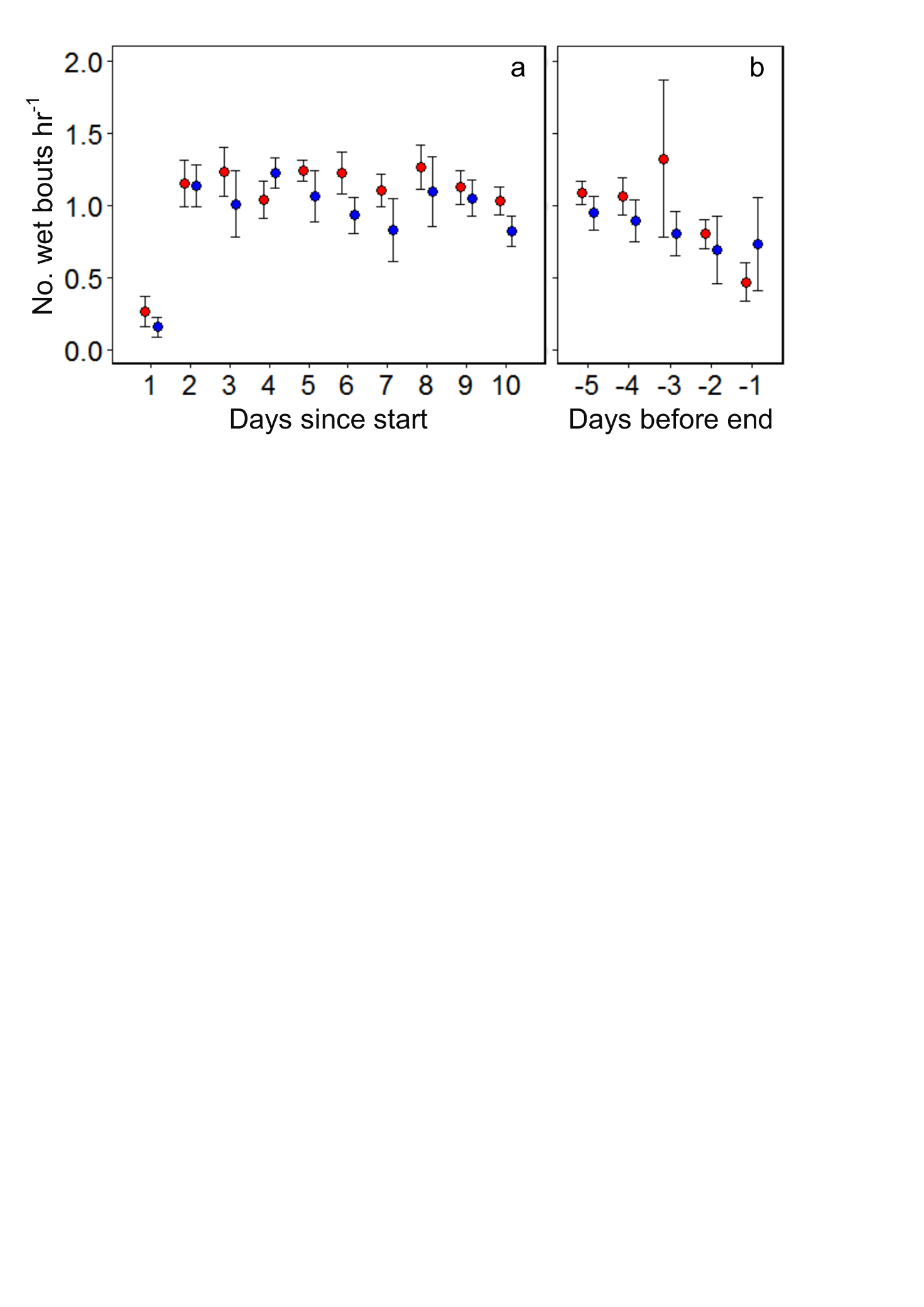


**Fig. S1.** The temporal distribution of wet bouts per hour summarised for each day during a) the first 10 days, and b) last five days of East (red) and South (blue) incubation foraging trips of Murphy’s petrels tracked with immersion loggers in 2015. Means ± SE of individual values are shown.


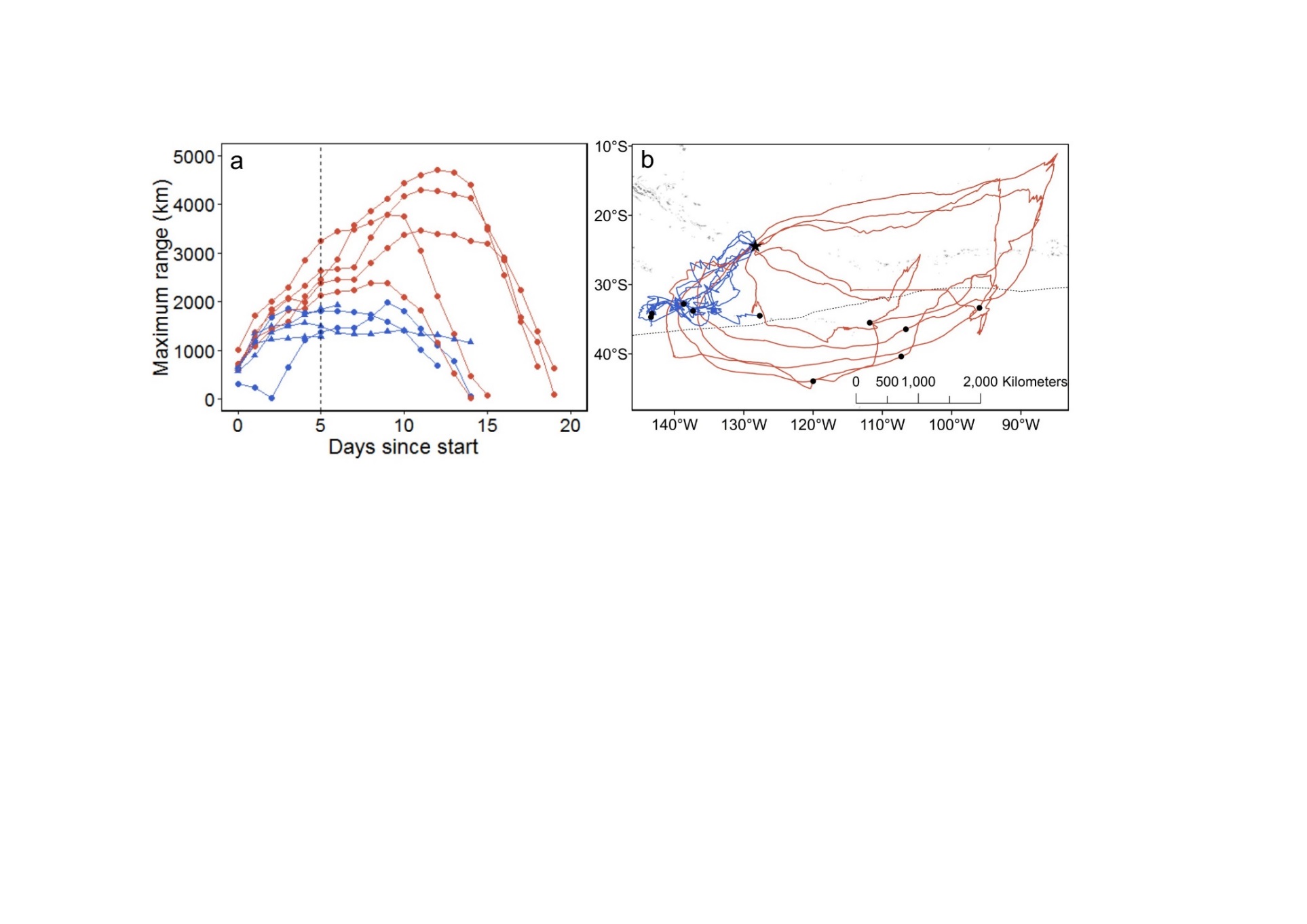


**Fig. S2**. The criteria used to classify trips as East (red) and South (blue). a) For each trip, we plotted the maximum range for each day, showing that by around four or five days into the trip (vertical dotted line), the maximum ranges were non-overlapping. Complete and incomplete trips are shown by dots and triangles, respectively. b) GPS tracks with the locations of birds on day five shown by black dots.


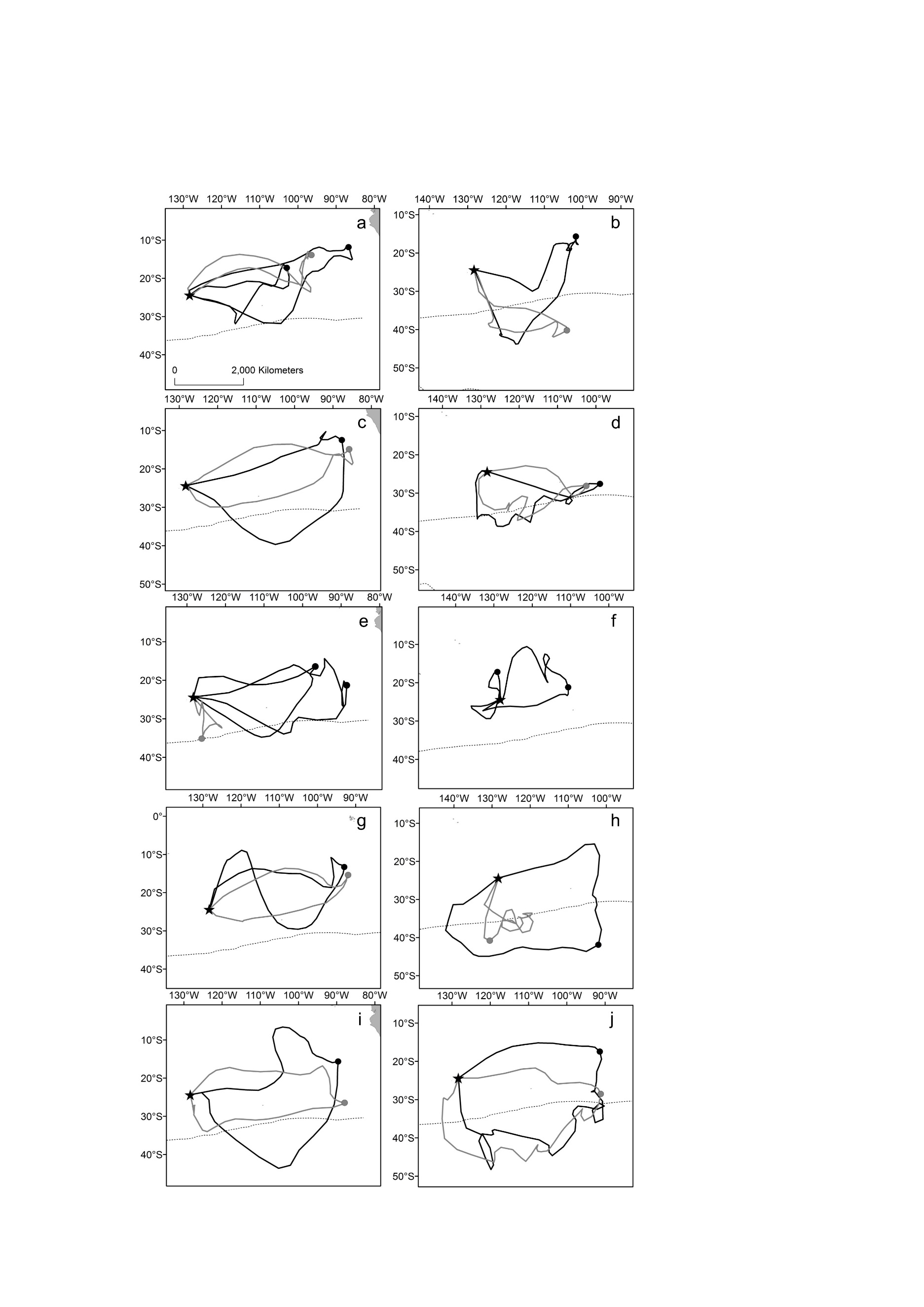


**Fig S3.** Repeat trips of 10 Murphy’s petrels tracked with geolocators in 2012 (black) and 2013 (grey), which are not shown in Fig. 6. Distal locations of trips are displayed as dots and the position of the Subtropical Front is shown with a dotted line. Note that the spatial extent of plots differs and that two individuals (a & e) were tracked for >1 trip in a given year.
